# Seroprevalence of influenza C and D virus infections among cattle in Japan

**DOI:** 10.1016/j.vas.2025.100468

**Published:** 2025-06-06

**Authors:** Kosuke Ohira, Kokoro Yokoe, Kaixin Li, Misa Katayama, Ayano Ichikawa, Akiko Takenaka-Uema, Wataru Sekine, Emi Takashita, Yasushi Muraki, Shin Murakami, Taisuke Horimoto

**Affiliations:** a Laboratory of Veterinary Microbiology, Graduate School of Agricultural and Life Sciences, University of Tokyo, Tokyo, Japan; b Influenza Virus Research Center, National Institute of Infectious Diseases, Tokyo, Japan; c Department of Microbiology, School of Medicine, Iwate Medical University, Iwate, Japan

**Keywords:** Influenza C virus, Influenza D virus, Cattle, Bovine respiratory disease complex, Seroprevalence

## Abstract

Although influenza C virus (ICV) and influenza D virus (IDV) circulate mainly among humans and cattle, respectively, recent studies in the USA have reported both viruses to be significantly related to the symptomatic bovine respiratory disease complex (BRDC), which causes economic losses in cattle farming. In this study, we conducted a serosurvey of ICV and IDV infections using cattle samples collected from five prefectures in Japan during 2020–2023. Hemagglutination inhibition testing indicated that the overall seroprevalence was 27.0 % for ICV and 48.5 % for IDV, the regional seroprevalence varied from 4.0 % to 36.9 % for ICV and from 10.8 % to 57.7 % for IDV, and the annual seroprevalence varied from 3.4 % to 23.2 % for ICV and from 21.8 % to 53.5 % for IDV. These results indicate that ICV and IDV are circulating widely among cattle in Japan, suggesting their possible involvement as the causative agents of BRDC.

## Highlights

• Both ICV and IDV are circulating widely among cattle in Japan.
• The seroprevalence is 27.0 % for ICV and 48.5 % for IDV.
• ICV infection was detected for the first time in cattle in Japan.
• Both ICV and IDV infections may be related to BRDC in Japan.

## 1 Introduction

Both influenza C virus (ICV) and influenza D virus (IDV), which belong to the *Orthomyxoviridae* family, contain a hemagglutinin-esterase-fusion glycoprotein (HEF) on their envelopes (Hause et al., 2013). HEF is involved in viral binding to cell receptors, fusion between the viral envelope and cell endosomal membrane, and viral release from cells via cell receptor destruction (Herrler & Klenk, 1991, Song et al., 2016). It is the primary target of antibody production in host immune responses against ICV and IDV infections. Although both viruses have been shown to utilize 9-*O*-acetyl sialic acid for cell receptors, they have different host ranges. ICV preferentially infects humans, especially children (Homma et al., 1982), and occasionally infects pigs (Guo et al., 1983) and dogs (Ohwada et al., 1987) but has limited pathogenicity, causing only mild respiratory symptoms. By contrast, IDV, which was first isolated from pigs with respiratory symptoms in the USA in 2011, has been shown to circulate mainly among cattle worldwide (Ducatez et al., 2015; Hause et al., 2014; Jiang et al., 2014; Murakami et al., 2016; Silva et al., 2022), with spillover to other animal species, including humans (Kwasnik et al., 2023).

The bovine respiratory disease complex (BRDC), which is caused by complicated viral and bacterial respiratory infections in hosts with a suppressed immune system due to stressors such as overcrowding, cold stimulation, and transportation, is the most economically devastating cattle disease worldwide (Grissett et al., 2015). Multiple retrospective metagenomic studies have revealed that IDVs are associated with symptomatic BRDC (Mitra et al., 2016; Ng et al., 2015). Experimental infection with IDV alone causes mild-to-moderate respiratory symptoms in cattle through targeting the upper and lower respiratory tracts. The virus can also be transmitted through direct and aerosol contact (Ferguson et al., 2016; Salem et al., 2019). In addition to IDVs, ICVs with high genome sequence homology to human ICVs have also been detected in cattle with respiratory symptoms in the USA (Zhang et al., 2018). Moreover, both ICV and IDV were detected at higher rates than other pathogens in nasal swabs from BRDC-afflicted cattle, suggesting that these influenza viruses may synergistically lead to BRDC (Nissly et al., 2020). No conclusive information currently exists on the pathogenicity of ICV infection alone in cattle.

We had previously reported the seroprevalence of IDV in cattle as well as IDV isolates of two lineages (D/Yamagata2016 and D/Yamagata2019) in Japan (Murakami et al., 2020). However, whether ICV circulates among cattle herds in the country remains unknown. Therefore, in this study, we conducted a serosurvey of ICV and IDV infections using cattle samples obtained from several prefectures in Japan.

## 2 Materials and methods

### 2.1 Viruses and cells

**Fig. 1:**
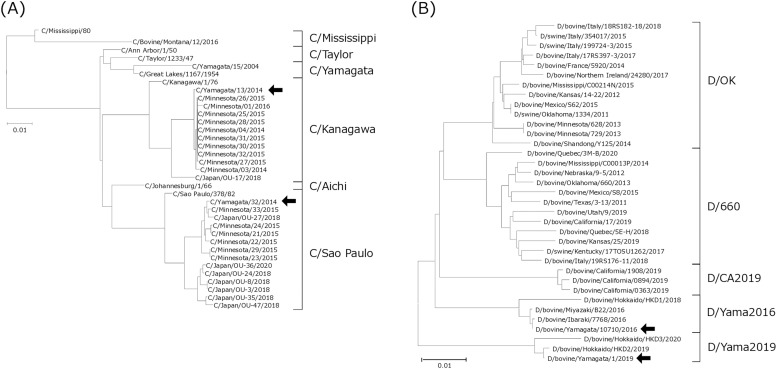
Phylogenetic tree for hemagglutinin-esterase-fusion glycoprotein (HEF) segments of (A) influenza C virus and (B) influenza D virus. Arrows indicate virus strains used in this study. Scale bars indicate nucleotide substitutions per site.

Japanese influenza virus strains of different phylogenetic lineages were used as antigens for the serological tests; namely, two ICV strains (C/Yamagata/13/2014 (C/Y13; C/Kanagawa lineage) and C/Yamagata/32/2014 (C/Y32; C/Sao Paulo lineage)) and two IDV strains (D/bovine/Yamagata/10710/2016 (D/Y16; D/Yama2016 lineage) and D/bovine/Yamagata/1/2019 (D/Y19; D/Yama2019 lineage)) (Fig. 1). All four strains were propagated in swine testis (ST) cells (CRL-1746; American Type Culture Collection, Manassas, VA, USA), which were maintained at 37 °C in Dulbecco’s modified Eagle’s medium (Fujifilm Wako Pure Chemical, Osaka, Japan) supplemented with 10 % fetal bovine serum. The virus-infected cells were maintained in Eagle’s minimum essential medium (Fujifilm Wako Pure Chemical) supplemented with 0.3 % bovine serum albumin and 0.5 µg/mL l-1-tosylamido-2-phenyl chloromethyl ketone-trypsin (Worthington, Lakewood, NJ, USA) and stored at –80 °C.

### 2.2 Cattle samples

From 2020 to 2023, we collected 2225 serum samples from cattle in five prefectures (Prefs. A to E) of Japan in accordance with legally required regular inspections and used the remainder for this serological survey. Prefs. A and B are located in the Kanto and Chubu regions, respectively, on the south side of central Japan; Prefs. C and D are located in Hokuriku and Chugoku regions, respectively, in the northwestern area; and Pref. E is located in the Kyushu region in the westernmost area of Japan. Samples were collected from apparently healthy Holstein and Japanese Black cattle.

### 2.3 Serological test

Hemagglutination (HA) inhibition (HI) assays were performed to detect serum antibodies against ICV and IDV. In the HI test, serum samples were treated with receptor-destroying enzyme (RDEII; Denka Seiken, Niigata, Japan) at 37 °C for 16 h, followed by heat inactivation at 56 °C for 30 min. The HI titer was expressed as the highest serum dilution ratio that completely inhibits the viral HA of 0.7 % turkey red blood cells by 4 HA units/25 µL after 30 min incubation at 21 °C with back-titration of the virus antigen. An HI titer of 1:40 or more was considered positive (Wood et al., 2012), and the positivity rate was calculated from the number of positive samples against at least one strain of ICV or IDV, respectively.

## 3 Results and discussion

Although both ICV and IDV are recognized as single serotypes, heterogeneous HEF antigenicity has been reported among viral phylogenetic lineages (Collin et al., 2015; Katayama et al., 2024; Matsuzaki et al., 2014; Odagiri et al., 2018). To assess the seroprevalence of ICV and IDV more accurately, we used two strains of each that are the major lineages circulating in Japan as antigens for the HI test. Of the 2225 cattle samples tested, 403 and 366 were positive for C/Y13 (18.1 % positivity) and C/Y32 (16.4 %) antibodies, respectively, whereas 988 and 577 samples were positive for D/Y16 (44.4 %) and D/Y19 (25.9 %) antibodies, respectively. In total, 601 and 1079 samples were positive for ICV (27.0 %) and IDV (48.5 %) antibodies, respectively (Table 1). These data indicate that the prevalence of IDV infection was equivalent to that reported in our previous study (Horimoto et al., 2016) and that ICVs are also circulating among cattle in Japan, albeit to a lower extent than IDV. Additionally, we detected 288 samples (12.9 %) that were positive for both ICV and IDV antibodies. Of these double-positive samples, 141 (49.0 %) showed HI titers equivalent to those of both viruses, minimizing antigenic cross-reactivity between ICV and IDV, and justifying infection with both viruses in these cows. Although this is the first report to show the presence of ICV in cattle in Japan, genetic detection and isolation of the virus are required to confirm its infection of these animals. Work to detect ICV genome sequences from nasal swabs of BRDC-afflicted cattle is currently underway. Nonetheless, it is most likely that ICV, in addition to IDV, may be one of the triggering agents of BRDC, as observed in the USA.

**Table 1:** Seroprevalence of ICV and IDV infections among cattle in Japan.

		ICV						IDV						ICV & IDV	
		C/Y13		C/Y32		Overall		D/Y16		D/Y19		Overall			
Pref.	sample #	positive #	positive %	positive #	positive %	positive #	positive %	positive #	positive %	positive #	positive %	positive #	positive %	positive #	positive %
Pref. A	105	6	5.7	6	5.7	12	11.4	21	20.0	17	16.2	23	21.9	2	1.9
Pref. B	1382	322	23.3	308	22.3	481	34.8	693	50.1	338	24.5	744	53.8	259	18.7
Pref. C	167	14	8.4	9	5.4	21	12.6	65	38.9	55	32.9	74	44.3	8	4.8
Pref. D	195	54	27.7	33	16.9	72	36.9	20	10.3	11	5.6	21	10.8	6	3.0
Pref. E	376	7	1.9	10	2.7	15	4.0	189	50.2	156	41.5	217	57.7	13	3.5
Total	2225	403	18.1	366	16.4	601	27.0	988	44.4	577	25.9	1079	48.5	288	12.9

Hemagglutination inhibition tests against C/Yamagata/13/2014 (C/Y13), C/Yamagata/32/2014 (C/Y32), D/bovine/Yamagata/10710/2016 (D/Y16), and D/bovine/Yamagata/1/2019 (D/Y19) were performed on serum samples collected in five prefectures (Pref.) in Japan.

From a regional perspective, Pref. D had the highest ICV-positive rate (36.9 %) and Pref. E the lowest (4.0 %), whereas Pref. E had the highest IDV-positive rate (57.7 %) and Pref. C the lowest (10.8 %) (Table 1). Therefore, the prevalence ratios of the two viruses varied depending on the prefecture, with ICV infections being more prevalent in Pref. D and IDV infections more prevalent in Pref. E. Instances of cattle transport between Prefs. A and E (∼10,000 km apart) and among Prefs. B, C, and D (also within that distance) were limited, which partly explains the different prevalence ratios of ICV and IDV infections between the regions. A similar situation was reported in a previous study, which showed that the prevalence of ICV and IDV infections varied among various states in the USA (Uprety et al., 2023). With regard to each sampling year, positive rates ranged from 3.4 % in 2022 to 32.2 % in 2020 for ICV and from 21.8 % in 2022 to 53.5 % in 2023 for IDV (Table 2). The geographic and periodic distributions of ICV and IDV infections in cattle fluctuate as a result of undefined complex factors, including breeding practices, animal movement, and environmental conditions.

**Table 2:** Annual seroprevalence of ICV and IDV infections among cattle in Japan.

		ICV						IDV					
		C/Y13		C/Y32		Overall		D/Y16		D/Y19		Overall	
Year	sample #	positive #	positive %	positive #	positive %	positive #	positive %	positive #	positive %	positive #	positive %	positive #	positive %
2020	1598	344	21.5	323	20.2	514	32.2	766	47.9	398	24.9	831	52.0
2021	497	53	10.7	37	7.4	75	15.1	186	37.4	147	29.6	206	41.4
2022	87	3	3.4	0	0	3	3.4	15	17.2	15	17.2	19	21.8
2023	43	3	7.0	6	14.0	9	20.9	21	48.8	17	39.5	23	53.5

Hemagglutination inhibition tests against C/Yamagata/13/2014 (C/Y13), C/Yamagata/32/2014 (C/Y32), D/bovine/Yamagata/10710/2016 (D/Y16), and D/bovine/Yamagata/1/2019 (D/Y19) were performed on serum samples collected between 2020 and 2023.

In conclusion, this study provided serological evidence that both IDV and ICV are circulating widely among cattle in Japan. Very few serosurveys of ICV infections in cattle have been reported in other countries (Salem et al., 2017). Further epidemiological and virological studies are required to clarify the correlations of ICV and IDV with the onset of BRDC in cattle as well as their zoonotic risk. Other serological tests, such as a viral neutralizing assay, should be performed to explore the false positives and negatives in HI assays to obtain more precise epidemiological data and to assess the zoonotic risk of ICV and IDV infections.

## Ethical statement

The animal experiments were approved by the Animal Experiment Committee of the Graduate School of Agricultural and Life Sciences, University of Tokyo (approved number: P16–300). All experimental procedures involving animals were conducted in strict accordance with relevant guidelines and regulations.

## CRediT authorship contribution statement

**Kosuke Ohira:** Writing – original draft, Visualization, Validation, Methodology, Investigation, Formal analysis, Data curation. **Kokoro Yokoe:** Investigation, Data curation. **Kaixin Li:** Investigation. **Misa Katayama:** Investigation. **Ayano Ichikawa:** Investigation. **Akiko Takenaka-Uema:** Investigation. **Wataru Sekine:** Validation, Investigation, Data curation. **Emi Takashita:** Resources, Methodology. **Yasushi Muraki:** Resources, Methodology. **Shin Murakami:** Supervision, Methodology, Investigation, Conceptualization. **Taisuke Horimoto:** Writing – review & editing, Supervision, Project administration, Methodology, Funding acquisition, Conceptualization.

## Declaration of competing interest

The authors declare that they have no known competing financial interests or personal relationships that could have appeared to influence the work reported in this paper.
